# Maternal Age-Related Depletion of Offspring Genetic Variance in Immune Response to Phytohaemagglutinin in the Blue Tit (*Cyanistes caeruleus*)

**DOI:** 10.1007/s11692-014-9301-8

**Published:** 2014-12-04

**Authors:** Szymon M. Drobniak, Anna Dubiec, Lars Gustafsson, Mariusz Cichoń

**Affiliations:** 1 Institute of Environmental Sciences, Jagiellonian University, ul. Gronostajowa 7, 30-387 Kraków, Poland; 2 Museum and Institute of Zoology, Polish Academy of Sciences, Warsaw, Poland; 3 Departament of Ecology and Genetics/Animal Ecology, Evolutionary Biology Center, Uppsala University, Uppsala, Sweden

**Keywords:** Heritability, Age, Immunocompetence, Blue tit, Genetic interaction

## Abstract

Studies examining age-specific patterns in genetic variance have focussed primarily on changes in the genetic variance within cohorts. It remains unclear whether parental age may affect the genetic variance among offspring. To date, such an effect has been reported only in a single study performed in a wild bird population. Here, we provide experimental evidence that the additive genetic variance (V_A_) observed among offspring may be related to parental age in a wild passerine—the blue tit (*Cyanistes caeruleus*). To separate genetic and environmental components of phenotypic variance in nestling body size and immune function we cross-fostered nestlings between pairs of broods born to young and old mothers and used an animal model to estimate V_A_. We show that the genetic variance in immune response to phytohaemagglutinin (PHA) and body weight among offspring depends on maternal age. V_A_ in response to PHA appeared to be lower among nestlings of older mothers. Such a tendency was not observed for tarsus length. We argue that the lower V_A_ may result either from depletion of additive genetic variation due to selection acting on parents across age classes or from environmental effects confounded with parental age. Thus, our study suggests that parental age may significantly affect estimates of quantitative genetic parameters in the offspring.

## Introduction

Evolutionary processes rely on the presence of additive genetic variance: evolutionary change is possible only if significant heritable variation in a phenotypic trait is present (Lynch and Walsh 1998). Substantial effort has been devoted to studying genetic variability and the interplay between genetic and environmental effects in shaping the evolution of quantitative traits (Ingleby et al. 2010; Nystrand et al. 2011; Wolinska and King 2009). Particular attention has been paid to genotype-by-environment interactions (GEIs), as they are regarded as a major force maintaining genetic variability in populations under natural selection (Lande and Shannon 1996; Roff 1997; Storfer 1996). However, environment is not the only factor that may influence the expression of genetic variance. Sex- or age-specific expression of genetic variance may also contribute to our understanding of mechanisms maintaining genetic variability in traits undergoing selection (Charlesworth and Hughes 2000; Hall et al. 2010; Seppala and Jokela 2010).

Sex-specific additive genetic variance (V_A_) has been reported in several studies (Drobniak et al. 2010; Jensen et al. 2003; Poissant et al. 2010). It may be present in the form of sex-specific heritabilities (e.g. Drobniak et al. 2010; Jensen et al. 2003; Weiss et al. 2006) and as non-existing or even negative cross-sex genetic correlations (Drobniak et al. 2010; Poissant et al. 2010). In contrast, parental age has rarely been considered as a factor influencing genetic variance. Such age-specific effects should be expected if specific genotypes survive across age classes, so different sets of alleles are transmitted by young and old parents. Age-related (within a specific individuals) changes in the breeding value have been demonstrated in a number of studies (e.g. Charmantier and Reale 2005; Wilson et al. 2007) (but see Brommer et al. 2010). However, it remains unclear whether parental age may affect genetic variance in the offspring.

Parental age constitutes an important determinant of the offspring fitness (see Liu et al. 2011 for a recent review). Offspring of older parents reproduce at a lower rate (great tit *Parus major*; Bouwhuis et al. 2010) and show shorter life expectancy (fruit fly *Drosophila melanogaster*; Moore and Harris 2003; but see also Priest et al. 2002—cockroach *Nauphoeta cinerea*). The mechanisms behind these age-specific effects are, however, poorly understood and clearly taxon-restricted. They are usually explained in terms of non-genetic age-specific parental effects (e.g. age-related reduction in the ability to provide sufficient parental care), but may also arise for genetic reasons (e.g. accumulation of mutations and age-related changes in genotypic interactions). Even if seemingly non-genetic, results of senescence may have a significant quantitative genetic basis, which may profoundly alter evolutionary dynamics of traits and thus always should be considered in a quantitative genetic framework (Charmantier et al. 2014). To our knowledge only three studies attempted to study whether parental age influences age-specific genetic variance. An increase in genetic variance of morphological traits of the offspring with increasing parental age has been suggested in laboratory populations of the fruit fly (*Drosophila melanogaster*; Beardmore et al. 1975) and in the guppy (*Poecilia reticulata*; Beardmore and Shami 1985). In contrast, lower genetic variance in age at first reproduction was observed among offspring of older fathers in a wild population of blue-footed boobies (*Sula nebouxii*; Kim et al. 2011). Thus, genetic mechanisms may be responsible for possible age-specific decline in offspring performance. More studies focusing on natural populations are however needed, in particular because patterns of age-specific heritabilities may substantially differ between wild and laboratory populations with reduced selection (Beardmore and Shami 1985; Kim et al. 2011). Moreover, studying the influence of parental age on the genetic variance and evolutionary potential may open a new perspective in quantitative genetics, as such effects have usually been neglected in quantitative genetics analyses.

Here we experimentally test whether maternal age may affect additive genetic variance observed among offspring in the blue tit (*Cyanistes caeruleus*). In our study, we estimate genetic variance in tarsus length, body weight and the immunological reaction to phytohaemagglutinin (PHA). These traits are often considered in quantitative genetics studies on birds and show moderate to high levels of additive genetic variance (Cichoń et al. 2006; Drobniak et al. 2010; Jensen et al. 2003; Kilpimaa et al. 2005; Merilä and Fry 1998; Pitala et al. 2009). These traits have also repeatedly been shown to influence reproductive success or survival and hence may constitute important selection targets (Alatalo and Lundberg 1986; Cichoń and Dubiec 2005; Garnett 1981; Møller and Saino 2004). In order to separate environmental and genetic variance we experimentally paired broods of females belonging to two distinct age classes and cross-fostered nestlings within those pairs. We analysed the resulting phenotypic data using an animal model (Henderson 1950, 1984; Kruuk and Hadfield 2007) which allows one to separate genetic and non-genetic sources of trait variance. We predict that additive genetic variance should differ between offspring mothered by young and old females. In contrast to the above-mentioned earlier studies we present rigorous analyses based on experimental age-based cross-fostering which provide a novel approach to studying age-specific genetic effects.

## Materials and Methods

### Study System and Field Procedures

We studied a wild population of blue tits on the Baltic island of Gotland (57°01′N 18°16′E), about 120 km off the eastern Swedish coast (see Pärt and Gustafsson 1989 for a detailed description of the study area). The population of blue tits on Gotland is characterised by relatively high return and recruitment rates to the breeding grounds (40 and 16 % respectively; own unpublished data), compared to continental populations. In this population, blue tits lay one clutch per season. Females lay on average 11 eggs (varying between 6 and 17 eggs). Young hatch after 2 weeks and fledge after the next 18–22 days. Individuals usually live up to 3 years, but individuals living 5–7 years have also been recorded.

Our study was performed over three consecutive years (2004–2006). Each year, from the end of April, we inspected nest-boxes regularly to locate blue tit nests. For each nest the number of eggs, date of laying and date of hatching (day 0) were recorded. Nestlings were uniquely marked by nail clipping (day 2) and later fitted with uniquely numbered aluminium rings (day 8). All nestlings were weighed at day 14th (electronic balance—Kern, Balingen, Germany, to the nearest 0.1 g) and measured for tarsus length (electronic calliper—Mitutoyo, Japan, to the nearest 0.01 mm).

To measure individual immune responsiveness to an unknown antigen we used delayed-type hypersensitivity reaction (Demas et al. 2011). To induce the reaction we injected PHA into the wing web of nestlings. PHA is a lectin derived from common bean seeds (*Phaseolus vulgaris*) that has a strong mitogenic effect on T lymphocytes (Goto et al. 1978). The hypersensitivity reaction involves cell-mediated, humoral and non-specific defense mechanisms (see Demas et al. 2011 for discussion), thus it may be considered as a general measure of readiness of immune system to fight antigens. 0.2 mg of PHA (Sigma Aldrich) suspended in 0.04 ml of saline was injected into the right wing web. The thickness of the wing web was measured thrice prior to and 24 h (±1 h) after the injection using a pressure-sensitive gauge micrometer (Mitutoyo, to the nearest 0.01 mm). All measurements were taken by the same person. The mean value of the three repeated measurements was used in further analyses. The level of hypersensitivity reaction was expressed as the intensity of swelling, i.e. the difference between the means of the first and post-24 h measurements.

Both parents were caught when feeding young between day 11th and fledging. Unringed birds were fitted with a uniquely numbered leg-ring. Tarsus length and body mass of all captured breeders was recorded. Age (first-year, henceforth young females; or older, henceforth old females) was determined according to the presence of a distinct moult limit between greater and primary wing coverts in individuals born the previous year (1 year old) or uniformly colored wing coverts in older individuals. Available age data indicate that majority of the older group were 3 years old individuals (~60 %), with a small proportion of 4-years old (~25 %) and ≥ 5-years-old females (~15 %). Of all females, only two were used twice in consecutive years; the remaining females are unique across all years.

**Fig. 1:**
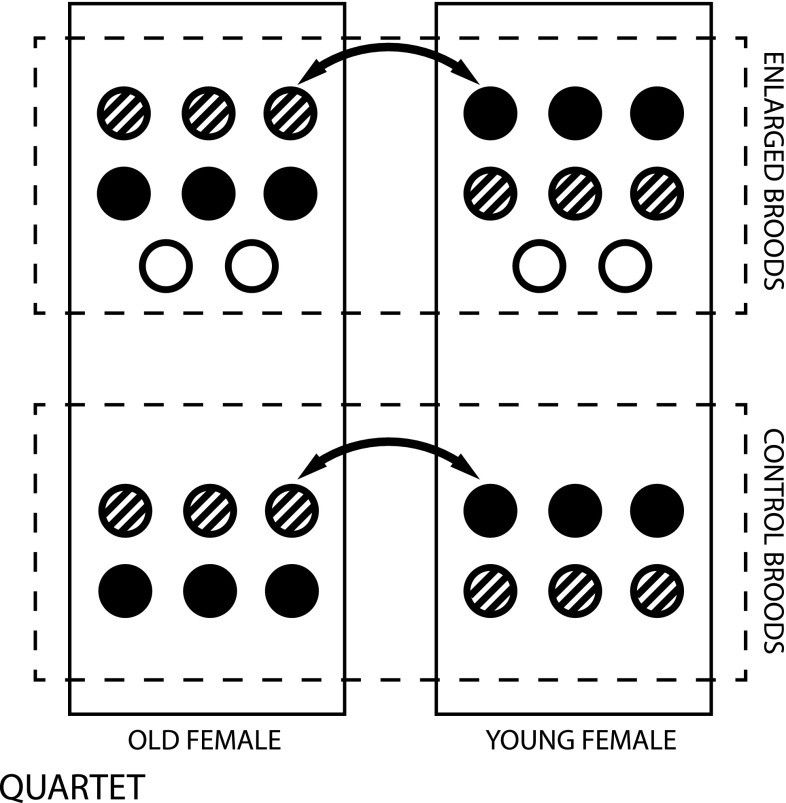
Schematic illustration of experimental design. *Solid*-*line* *rectangles* depict individual nests, between which nestlings where cross-fostered (*arrows*). *Full* and *dashed circles* depict individual experimental nestlings, *open circles* depict donor nestlings used in the brood-size manipulation experiment. Note that for clarity only six experimental nestlings are depicted for each clutch, a number that differed depending on the original clutch-size

In our study we matched newly hatched broods of young females and old females in quartets containing two young-mother’s nests and two old-mother’s nests. Nests within a quartet were matched by date of hatching (±1 day) and number of nestlings (±1 nestling). Two days after hatching we cross-fostered nestlings following a split-brood design, such that half of the nestlings were exchanged inside pairs containing a nest of the young female and a nest of the old female (Fig. 1). The cross-fostering allowed us to separate additive genetic and post-hatching brood environment effects (Kruuk and Hadfield 2007). Two randomly selected nests within a quartet (one nest of a young female and one of an old female) were subjected to brood-size manipulation (being enlarged by three nestlings coming from a nest not used in the quartets). Brood size manipulation was considered in another study. However, as it is crossed with age-specific groups, the effect of brood manipulation should not be confounded with the effect of mother’s age (Fig. 1). Thus, the brood size manipulation is included in our statistical analyses to account for possible influence of the brood enlargement, but we do not focus on this effect throughout the paper since in our system brood enlargement seems to have no effect on the genetic variance in the responsiveness to PHA (Drobniak et al. 2010).

In total, 25 quartets were created, evenly distributed across years. Our analyses comprise 1,092 nestlings. In 2004 the hypersensitivity reaction was not measured and hence only 2005 and 2006 were considered in the analyses of PHA response. In total, 485 nestlings from 18 quartets were tested for the PHA response.

### Quantitative Genetic Analyses

#### Data Quality and Preparation

We applied an animal model (a type of a linear mixed-effects model; Kruuk 2004) with age-dependent (co)variance structure to estimate genetic and environmental effects on PHA response, body mass on the 14th day and tarsus length. The models were fitted using ASReml-R 3.1 (Butler 2009) implemented in R (version 3.0.14; R Core Team 2014).

Animal model is a special case of the linear mixed model that uses all available genealogical information about relationship between individuals (i.e. a pedigree) to estimate the contribution of additive genetic effects to the total phenotypic variance (Henderson 1950, 1984; Kruuk 2004). Initially, our pedigree included 1,317 individuals (offspring and their parents) from 3 cohorts (offspring from the years 2004, 2005 and 2006, plus their parents from generation preceding the year 2004). However, in the studied population, about 20 % of offspring recruit in the following years and about 40 % of adult individuals are observed more than once. Reduced recruitment is a common issue in open, wild populations that are not controlled with respect to the breeding design and therefore provide data with many missing links in the pedigree. Also, not all nestlings were measured for all analyzed traits. Thus, the effective number of individuals contributing to the estimation of additive genetic effects was reduced. After cleaning and pruning the pedigree using the *pedantics* R package (Morrissey and Wilson 2010), the number of individuals contributing to the estimation of genetic variance in body weight and tarsus length was 1,090, with 874 maternities, 872 paternities, mean maternal sibship size of 7.8 and mean paternal sibship size of 8.2. For PHA response 355 individuals contributed to the estimation of additive genetic variance, with 278 maternities, 278 paternities, mean maternal sibship size of 6.9 and mean paternal sibship size of 6.95. Our analyses are based on nestling phenotypes and since all nestlings were part of a large-scale cross-fostering procedure brood effects are not confounded with genetic effects in our analyses.

*Animal models* Study year (2004, 2005, 2006), maternal age (young vs. old), and brood size manipulation (enlarged vs. control) were defined as fixed explanatory variables. To test for possible confounding influence of the interaction between maternal age and experimental treatment it was included in all initial models, but it appeared non-significant in all analyses (*P* > 0.5 in all cases), thus we do not consider this interaction in the presented results. Additional fixed effects were included in specific models. For body weight and tarsus length we included sex, to take into account a well-documented size dimorphism in the studied species (Blondel et al. 2002). The analysis of body weight included also tarsus length as a covariate, to correct weight measurements for the structural body size. Finally, in the analysis of PHA response we included body mass as a covariate, which is a usual practice accounting for the correlation between the body weight and the PHA-related skin swelling (Alonso-Alvarez and Tella 2001).

In addition to fixed effects, we modeled a number of random effects in all animal models: additive genetic effect (*V*_*A*_), nest-of-origin (termed origin henceforth), nest-of-rearing (termed rearing henceforth) and quartet identity. Interpretation of the non-genetic random effects is as follows: (1) origin estimates common origin variance, especially early maternal and common-environment effects (Lynch and Walsh 1998); (2) rearing effect explains how much of the total variance comes from a shared rearing environment; (3) quartet identity accounts for possible variance between quartets, emerging primarily due to differing hatching dates and other environment-related sources. To enable maternal age-specific genetic effects, additive genetic effects were modeled in the form of a 2 × 2 square covariance matrix, with two age-specific variances on its diagonal. Cross-fostering decouples brood (common environment) and genetic effects and thus in our analysis it was possible to estimate genetic covariance between two maternal age groups.

#### Testing of Fixed and Random Effects

Fixed effects were tested using an adjusted Wald statistics (Butler 2009). Since random effects in our study system have implicitly hierarchical structure, significance of all random effects and age-related differences in genetic and residual variances were tested using likelihood-ratio test (LRT), using a sequence of models of increasing complexity (Pinheiro and Bates 2000). Likelihood-ratios for testing variances were assumed to follow a Chi squared distribution with *df* = 1, as always only one parameter more was estimated in the more complex model. Self and Liang (1987) recommend a modified mixture Chi squared distribution (a mixture of *χ* ^2^ with *df* = 1 and *df* = 0) for testing variances (for which the null-hypotheses are at the boundary of parameter, effectively resulting in *P* values for the test equal half of the *P* value with *df* = 1)—however, using Chi squared distribution with *df* = 1 is more conservative.

The most important part of the random effects structure—the maternal age-dependent genetic (co)variances—was tested by fitting a series of complex models. We predict that—under the null hypothesis—variances in two maternal age classes are equal and genetic correlation between these classes is equal to one. Verification of these hypotheses required the following models (we provide also the number of parameters describing the random effects part of the model, including all estimated random effects): (1) model assuming no differences in *V*_*A*_ related to maternal age (five parameters estimated); (2) model with maternal age-dependent *V*_*A*_ (*V*_*A*1_ ≠ *V*_*A*2_) and genetic correlation between maternal age classes fixed at unity (r_xage_ = 1; six parameters estimated); (3) model with *V*_*A*1_ ≠ *V*_*A*2_ and unconstrained covariances (−1 ≤ *r*_*xage*_ ≤ 1; seven parameters estimated); (4) model with *V*_*A*1_ ≠ *V*_*A*2_ and *r*_*xage*_ = 1, but with residual variances differing between maternal age classes (to account for the possibility that heterogeneous residual variances might generate heterogeneous *V*_*A*_; seven parameters estimated). Models were compared in the order specified in Table 1. Fixing cross-age correlations at unity represents the null hypothesis assuming that females in different age classes share identical genetic background and thus we predict full genetic correlation between them (Lynch and Walsh 1998).

**Table 1:** Means and variances of all analyzed traits, split between young and old genetic mothers

Trait	Genetic mother	Mean	Variance	CV
Tarsus length (mm)	Young	16.17	0.42	0.12
	Old	16.16	0.43	0.12
Body weight (g)	Young	10.59	1.01	0.12
	Old	10.62	1.05	0.12
PHA response (mm)	Young	0.82	0.10	0.40
	Old	0.74	0.04	0.26

In addition to genetic effects, heterogeneous covariance matrices, with cross-age correlations fixed at unity, were fitted to the nest-of-rearing and nest-of-origin effects. These models were compared with a simpler model with heterogeneous variances in the additive genetic effect to make sure that brood and early parental effects (decomposed into rearing and origin nest effects by cross-fostering) do not inflate/bias our estimates of *V*_*A*_.

For all models, narrow-sense heritabilities (*h* ^2^) of traits were calculated. To calculate *h* ^2^ we divided respective *V*_*A*_ by the sum of all variance components (Lynch and Walsh 1998). Standard errors of heritabilities were estimated using the delta-method (Lynch and Walsh 1998). Final models did not support genetic correlations between maternal age groups significantly lower than unity and thus we do not provide estimates of genetic correlations.

### Technical Notes

Our estimates of genetic variance might be biased as offspring from one nest-of-origin might not be full siblings. In our population, about 20 % of nests contain extra-pair young (usually one nestling per nest; unpublished data from years not included in this study), resulting in overall prevalence of extra-pair young of approx. 4 %. Such a level of extra-pair paternity should not strongly bias estimates of genetic variance, as predicted from simulation models (Charmantier and Reale 2005). We have performed similar simulations, assuming the level of pedigree uncertainty similar to this observed in our population; these simulations indicate that small inconsistencies in the pedigree do not affect significantly even more complex estimated (co)variance structures (bias in differences in heritabilities and genetic correlations do not exceed 5 %). Moreover, effects we have observed in simulated data bias observed differences in heritabilities downwardly and hence act conservatively. Recent meta-analysis also suggests that bias in quantitative genetic studies introduced by errors in the pedigree may be less substantial than previously expected (Postma 2014). Finally, distribution of extra pair young shows no association with female age classes (*χ*_*df*=1_^2^ = 1.11, *P* = 0.29, based on data from the same population, years 2009–2011) and thus it is not likely to lead to the observed effect of maternal age. Other sources of pedigree error (such as intra-species brood parasitism) are not observed in our population.

Paternal age might contribute to the observed patterns if males and females in the studied population mate assortatively with respect to individual’s age. In such a case effects of paternal age might be inseparable from the effects of maternal age. However, this should not be the case in our population. Based on the available complete (i.e. both parents known) data on breeding pairs in the population in years 2004–2006 there is no evidence for assortative mating according to age (194 unique breeding pairs, test for assortativity according to age (two age classes): *χ*_*df*=1_^2^ = 0.33, *P* = 0.57). Moreover, experimental groups were formed with respect to maternal age as females can be more easily caught (on incubation) prior to hatching.

## Results

Maternal age did not have any significant effect on any of the traits analyzed (body weight: *P* = 0.61, tarsus length: *P* = 0.58, PHA response: *P* = 0.74; Table 1, Appendix Table 4) but was retained in the model as it was used to structure covariance matrices for genetic and residual variance. Experimental brood manipulation affected all traits (Appendix Tables 4, 5): offspring in experimentally increased broods were lighter (*P* < 0.001) and had shorter tarsi (*P* = 0.06). There was a trend of higher response to PHA in enlarged broods but it was not significant (*P* = 0.12). Models that attempted to split sources of phenotypic variation between maternal age groups and experimental groups (a 4 × 4 covariance matrix) had problems reaching convergence, which likely resulted from complex nature of fitted models. We therefore do not discuss experimental manipulation further in terms of partitioning of variance components. Experimental manipulation was not confounded with maternal age groups (Fig. 1) and thus it cannot bias conclusions related to age—however, in all models considering age-specific effects on variance components experimental treatment is included as a fixed explanatory variable.

All random effects (except for quartet for tarsus length and additive genetic effect in body mass) appeared significant based on the LRT. Particularly, in tarsus length and PHA response we found a significant additive genetic component (Table 2).

**Table 2:** Likelihood-ratio tests of variance components

Model^a^	No.	Test	log(L)	Δlog(L)	P	Significance of…
*Tarsus length*						
E	1	–	−56.21			
E Q	2	2 versus 1	−56.21	0	–	Experimental quartet effect
E R	**3**	**3 versus 1**	**45.32**	**101.52**	**<0.001**	Nest-of-rearing effect
E R O	**4**	**4 versus 3**	**69.67**	**24.35**	**<0.001**	Nest-of-origin effect
E R O A	**5**	**5 versus 4**	**72.55**	**2.88**	**0.008**	Additive genetic effect (*V*_*A*_)
E R O Age(A)	6	6 **versus** 5	73.14	0.58	0.146	Age dependence of *V*_*A*_
Age(E) R O A	7	7 versus 5	73.34	0.79	0.103	Age dependence of residual variance (*V*_*E*_)
Age(E) R O Age(A)	8	8 versus 7	73.35	0.01	0.499	Test for confounding effect of *V*_*E*_ on *V*_*A*_
		8 versus 6	73.35	0.78	0.103	
*Body mass*						
E	1	–	−302.24			
E Q	**2**	**2 versus 1**	−**300.09**	**2.14**	**0.038**	Experimental quartet effect
E Q R	**3**	**3 versus 2**	−**156.61**	**143.47**	**<0.001**	Nest-of-rearing effect
E Q R O	**4**	**4 versus 3**	−**134.18**	**22.43**	**<0.001**	Nest-of-origin effect
E Q R O A	5	5 **versus** 4	−133.27	0.91	0.061	Additive genetic effect (*V*_*A*_)
*PHA response*						
E	1	–	360.21			
E Q	**2**	**2 versus 1**	**367.87**	**7.61**	**<0.001**	Experimental quartet effect
E Q R	**3**	**3 versus 2**	**415.13**	**47.3**	**<0.001**	Nest-of-rearing effect (*V*_*R*_)
E Q R O	**4**	**4 versus 3**	**430.06**	**14.94**	**<0.001**	Nest-of-origin effect (*V*_*O*_)
E Q R O A	**5**	**5 versus 4**	**431.64**	**1.57**	**0.003**	Additive genetic effect (*V*_*A*_)
E Q R O Age(A)	**6**	**6 versus 5**	**447.96**	**16.33**	**<0.001**	Age dependence of *V*_*A*_
Age(E) Q R O A	7	7 versus 5	431.65	0.01	0.499	Age dependence of residual variance (*V*_*E*_)
Age(E) Q R O Age(A)	8	8 versus 6	448	0.04	0.479	Test for confounding effect of *V*_*E*_ on *V*_*A*_
		**8 versus 7**	**448**	**16.35**	**<0.001**	Age dependence of *V*_*A*_ in presence of age-dependent *V*_*E*_
E Q R O Age(A)^b^	9	9 versus 6	448.02	0.06	0.485	Cross-age genetic covariance lower than unity
E Q Age(R) Age(O) Age(A)	10	10 versus 6	449.09	2.77	0.09	Test for confounding effect of *V*_*R*_ and *V*_*O*_ on *V*_*A*_
E Q Age(R) O Age(A)	11	11 versus 6	449.08	2.76	0.09	Test for confounding effect of *V*_*R*_ on *V*_*A*_
E Q R Age(O) Age(A)	12	12 versus 6	447.95	0.01	0.99	Test for confounding effect of *V*_*O*_ on *V*_*A*_

Bold indicates significant results in model comparisons

log(L), logarithm of likelihood; Δlog(L), difference in log-likelihoods of the more complex and simpler model; *P*, significance of the random effect added in the more complex model, as compared to the simpler model; Test, which models were compared. The last column provides the interpretation of each model comparison

^a^Terms in models are labelled in the following way: E, residual variance; Q, quartet; R, nest of rearing; O, nest of origin; A, additive genetic effect; Age(X), (constrained) age-dependent covariance matrix is fitted (cross-age correlations constrained to unity for A and zero for E)

^b^Resulting covariance matrix is unconstrained (covariance is estimated)

**Fig. 2:**
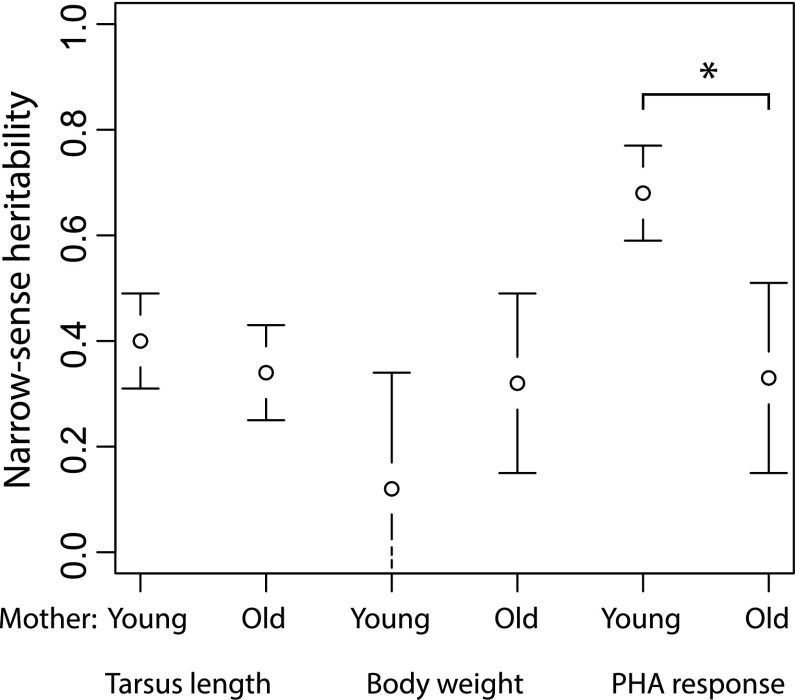
Age-specific differences in heritabilities (with their SE’s) of tarsus length, body mass and PHA response. Values for body mass and tarsus length were extracted from unsupported age-specific models to allow direct comparisons with PHA response

Age-specific genetic variances were observed in PHA (Tables 2 and 3). *V*_*A*_ in this trait appeared lower among old mothers’ offspring compared to young mothers’ offspring in case of (Table 3; Fig. 2). Age specific residual variances in this trait were not supported (all model comparisons: *P* > 0.1). There was also no evidence for age specific variance related to nest-of-rearing (*P* = 0.09) and nest-of-origin (*P* = 0.99), indicating that permanent environmental effects do not depend on maternal age. Overall trait variances closely matched results from animal models (Table 1): total variance in PHA response was lower in offspring of old mothers.

**Table 3:** Variance estimates and proportions of total phenotypic variance explained by relevant random effects ± SE from mixed-effects models

Trait	Additive genetic variance	Nest-of-origin variance	Nest-of-rearing variance	Experimental quartet variance	Residual variance
Body mass	0.28 ± 0.11 0.29 ± 0.11	<0.00001 <0.00001	0.38 ± 0.10 0.40 ± 0.07	<0.00001 <0.00001	0.29 ± 0.07 0.30 ± 0.08
Tarsus length	0.15 ± 0.04 0.37 ± 0.09	<0.00001 <0.00001	0.11 ± 0.03 0.27 ± 0.05	0 0	0.15 ± 0.02 0.36 ± 0.07
PHA response (young mother)	0.06 ± 0.01 0.68 ± 0.09	<0.00001 <0.00001	0.01 ± 0.004 0.10 ± 0.04	0.001 ± 0.003 <0.000001	0.02 ± 0.005 0.21 ± 0.07
PHA response (old mother)	0.01 ± 0.008 0.33 ± 0.18	<0.00001 <0.00001	0.01 ± 0.004 0.22 ± 0.08	0.001 ± 0.003 <0.000001	0.02 ± 0.005 0.45 ± 0.14

Variance components are provided as top values and respective proportions as bottom values. For components restricted by *ASReml* at the parameter space boundary (variances close to zero) we skip the standard error

*V*_*A*_ differences translated directly into heritability differences. Heritability of PHA response was higher among offspring of young (heritability ± SE: *h* ^2^ = 0.68 ± 0.09, Fig. 2) compared to old mother’s offspring (*h* ^2^ = 0.33 ± 0.18, Fig. 2). In tarsus length there were no maternal age-dependent differences in heritability (*h* ^2^ = 0.38 ± 0.08). In body mass heritability was non-significant (*h* ^2^ = 0.29 ± 0.12).

## Discussion

We found significant additive genetic variance in tarsus length and PHA response. After correcting for body size, we found also evidence for genetic variance in body mass, however it appeared non-significant. Our estimates of narrow sense heritabilities are similar to estimates reported elsewhere (Cichoń et al. 2006; Kruuk et al. 2001; Merilä and Fry 1998). More importantly, we demonstrated that maternal age is an important factor contributing to the complex picture of genotypic interactions. We found that additive genetic variance of immune response to PHA among offspring mothered by old females was substantially lower compared to the offspring of young females. In contrast, we found no cross-maternal-age differences in *V*_*A*_ with respect to tarsus length.

Although evidence for interaction of parental age and genetics effects is scarce, genetic variances and genetic correlations depending on parental age have been already reported in laboratory populations of fruit flies and guppies (Beardmore et al. 1975; Beardmore and Shami 1985). Those studies demonstrated higher genetic variances among offspring of older parents and argued that this might represent ageing processes, in line with the predictions of the mutation accumulation hypothesis (Charmantier et al. 2014; Medawar 1952). However, interpretation of their results is difficult due to the fact that parental age is confounded with grand-parental age in their experimental design. Also, the controlled laboratory rearing conditions may not be appropriate to study age-specific heritabilities (Charmantier et al. 2014). Selective forces resulting from environmental heterogeneity certainly shape the genetic structure of natural populations, while in the laboratory populations selection is usually relaxed and does not reflect conditions under which a species had evolved. To our knowledge, only one study reported parental age to affect genetic variance in a wild bird population (Kim et al. 2011). It showed a significant decrease in genetic variance of age at first reproduction with respect to paternal age, but failed to detect any effects of maternal age. In this context, our study is complementary to Kim et al. (2011). However, our analyses rely on experimental data in which variation in parental age was a priori experimentally manipulated by matching broods of young and old females and cross-fostering nestlings to account for any confounding effects of rearing environment. It is an important advantage as environmental and early post-hatching parental effects might potentially be confounded with parental age if environmental effects are not randomly distributed across age classes leading to biased estimates of genetic variances. Cross-fostering also enables us to exclude early post-natal and maternal effects as likely drivers of observed differences: all such effects would inflate estimates of nest-of-origin variance which in our study remained consistently low, and homogenous between maternal age groups.

Our results add to the growing evidence that the genetic architecture of wild populations is complex (Jensen et al. 2003; Nystrand et al. 2011; Poissant et al. 2010; Seppala and Jokela 2010; Wilson et al. 2007), and demonstrates the potential importance of considering parental age in quantitative genetic studies, especially when applying analyses based on full-sib comparisons without employing animal model. The estimates of *V*_*A*_ presented here clearly suggest that younger mothers may contribute more to the overall genetic variation observed in the population in specific traits. As a consequence, the response to selection may be stronger among the offspring of younger mothers. In many animal taxa clear age-structuring is observed in populations (e.g. Laws et al. 2010; Pelletier et al. 2012; Soulsbury et al. 2011). Thus, any changes in age-structure of a reproducing population may alter its evolutionary trajectory and effective response to selection in cohorts expressing more genetic variance would dominate overall evolutionary change in population. More specific predictions of evolutionary dynamics of such systems are likely to be challenging as both offspring phenotypes and parental traits (age) are taken into account. Univariate breeder’s equation may be inappropriate in such situations (McAdam et al. 2014) which motivates further detailed simulation studies.

An intriguing question on the origin of the observed patterns of age-specific genetic variances arises. Offspring of older parents may become more genetically uniform if specific genotypes are selectively removed from the population across age classes. In our population, survival rate from 1 to 2 years of age does not exceed 40 % (Podmokła et al., in preparation). Thus, there is potential for selection to operate. Immune function have repeatedly been shown to predict subsequent survival and reproductive success (Alatalo and Lundberg 1986; Møller and Saino 2004; Norris and Evans 2000). In particular, data gathered in the studied population support the presence of significant selection acting on immunocompetence measured by PHA response (Cichoń and Dubiec 2005). Unfortunately data gathered in the current study do not allow for direct estimation of selection gradients and thus selection can be treated only as one of possible mechanisms. Moreover, direct selection (suggested by Cichoń and Dubiec 2005) does not seem to be supported by our data as we have observed no significant difference in the mean PHA response in offspring between young versus old mothers. Certainly, this doesn’t rule out stabilizing selection, however more in-depth genetic analyses are required to support selective explanation.

Even in the absence of any selection acting on a trait, it’s quantitative genetics may be substantially altered by environmental conditions experienced by individuals. Numerous studies have provided evidence, that environment may influence levels of observed genetic variance (Gienapp and Brommer 2014; Hallsson and Bjorklund 2012; Hoffmann and Merila 1999; Ingleby et al. 2010; King et al. 2011; Nystrand et al. 2011; Wolinska and King 2009). Such mechanism would be possible in the presented case as often first-time breeders are less successful in caring for their young and securing high-quality habitats for them (Angelier et al. 2006) and thus would provide them with markedly different rearing environment. Such environmental heterogeneity might generate genotype-by-environment interactions (G × E) and result in the observed age-specific patterns of *V*_*A*_, particularly because immunocompetence strongly depends on multiple aspects of parental care (Ilmonen et al. 2003; Moreno et al. 1999; Saino et al. 1997). Another source of maternal age-related variation in environments experienced by nestlings include varying success of young and old females in securing high quality males. Older females—as more experienced—often tend to be more choosy or base their mate choice on different male characteristics than younger ones (Candolin 2003; Jouventin et al. 1999). This process alone could provide offspring with different “paternal” rearing environments, triggering interactions of genetic effects with varying experienced conditions. In either of these explanations female age has to be associated with some environmental features or father’s traits which points to studies exploring such associations as valuable extensions of our findings.

The abovementioned explanations are potentially universal in terms of affected traits, but our study may be specifically limited because of the choice of only one immunocompetence metric we have measured. Immune function is a complex multidimensional trait, thus conclusions drawn from a single measure of immune response to an artificial antigen should be taken with caution. In addition, the use of hypersensitivity reaction to PHA as a measure general immune function has been criticized by some authors (Demas et al. 2011; Sarv and Horak 2009; Vinkler and Albrecht 2011; Vinkler et al. 2012). However, the PHA test is commonly used and a number of studies reported significant heritability of reaction to PHA and more importantly is shows a significant correlation with a number of traits considered to be a fitness proxies, such as survival and recruitment (Cichoń and Dubiec 2005; Møller and Saino 2004). Here we provide analyses that lead to an important insight into the understanding of complex structure of quantitative traits and do not aim at generalizing our result concerning immune response to PHA as being representative for a proxy of immune function as a whole.

To conclude, our study provides the first experimental evidence that additive genetic variance observed among offspring depends on maternal age. In age-structured populations such genetic heterogeneity may result in different age classes contributing differently to the overall genetic variation of the population and age-specificity of response to selection.

## Appendix

See Tables 4 and 5.

**Table 4:** Tests of fixed effects for all traits analyzed

Effect	Wald statistic	*P*
*Tarsus length*		
Brood-size manipulation	3	0.068
Female age	0	0.545
Year	33	<0.001
Sex	10	<0.001
*Body weight*		
Brood-size manipulation	34.92	<0.001
Female age	2.36	0.12
Year	0.27	0.6
Sex	46.1	<0.001
Tarsus length	28.67	<0.001
*Response to PHA*		
Brood-size manipulation	1.91	0.16
Female age	0.29	0.59
Body mass	24.63	<0.001

**Table 5:** Fixed effects estimates with their standard errors from all models considered

Effect	Estimate	SE
*Tarsus length*		
Intercept	16	0.11
Brood-size manipulation	−0.25	0.09
Female age	−0.02	0.06
Year (2005)	0.56	0.13
Year (2006)	0.09	0.15
Sex	−0.25	0.09
*Body weight*		
Intercept	8.88	0.98
Brood-size manipulation	−1.16	0.2
Female age	0.17	0.12
Year (2005)	−60.24	96.29
Year (2006)	−72.64	115.51
Sex	0.43	0.22
Tarsus length	0.12	0.02
*PHA response*		
Intercept	−3.67	1.24
Brood-size manipulation	0.26	0.18
Female age	−0.11	0.2
Body weight	0.35	0.07
